# Fast segmentation of anterior segment optical coherence tomography images using graph cut

**DOI:** 10.1186/s40662-015-0011-9

**Published:** 2015-01-22

**Authors:** Dominic Williams, Yalin Zheng, Fangjun Bao, Ahmed Elsheikh

**Affiliations:** Ocular Biomechanics and Biomaterials Group, School of Engineering, University of Liverpool, Brownlow Hill, Liverpool, L69 3GH UK; Department of Eye and Vision Science, University of Liverpool, 3rd Floor, UCD Building, Daulby Street, Liverpool, L69 3GA UK; School of Optometry and Ophthalmology and Eye Hospital, Wenzhou Medical University, No. 270, Xueyuanxi Road, Wenzhou City, Zhejiang Province 325027 China

**Keywords:** Optical coherence tomography, Anterior segment, Image segmentation, Cornea, Graph cut, Shape prior

## Abstract

**Background:**

Optical coherence tomography (OCT) is a non-invasive imaging system that can be used to obtain images of the anterior segment. Automatic segmentation of these images will enable them to be used to construct patient specific biomechanical models of the human eye. These models could be used to help with treatment planning and diagnosis of patients.

**Methods:**

A novel graph cut technique using regional and shape terms was developed. It was evaluated by segmenting 39 OCT images of the anterior segment. The results of this were compared with manual segmentation and a previously reported level set segmentation technique. Three different comparison techniques were used: Dice’s similarity coefficient (DSC), mean unsigned surface positioning error (MSPE), and 95% Hausdorff distance (HD). A paired t-test was used to compare the results of different segmentation techniques.

**Results:**

When comparison with manual segmentation was performed, a mean DSC value of 0.943 ± 0.020 was achieved, outperforming other previously published techniques. A substantial reduction in processing time was also achieved using this method.

**Conclusions:**

We have developed a new segmentation technique that is both fast and accurate. This has the potential to be used to aid diagnostics and treatment planning.

## Background

The human eye is a remarkable pressurised organ and its biomechanical properties are essential in maintaining its functionality. There has been an increasing interest in modelling the biomechanics of the eye, as this will improve our understanding and management of eye disease. When carrying out biomechanical modelling of the eye, one of the aims is to be able to produce patient specific models so that the eyes of individual patients can be simulated providing important information towards personalised medicine. In order to produce patient specific models, geometry of the eyes in particular the cornea is required. A technique that can be used to gain such information on the cornea is optical coherence tomography (OCT) imaging. OCT is widely used in retinal imaging [1] and has increasing uses in imaging the anterior segment [2].

Anterior segment OCT (AS-OCT) allows the resolution of anterior and posterior surfaces of the entire cornea. This allows accurate measurement of the thickness and volume of the entire cornea, as well as the anterior chamber biometry such as its angle and depth. It has several important medical applications from contact lens fitting, diagnosis and clinical evaluation, surgical planning and monitoring, to monitoring patients with eye pathologies [3-5]. In particular, obtaining accurate topography information of the anterior segment using this technique would also allow construction of patient-specific models for biomechanical modelling of the human eye [6]. A potential use of biomechanical modelling is for studying patients with keratoconus [7,8]. Keratoconus causes a deformation of the cornea due to change in stiffness of parts of the cornea. Currently, surgical treatment is limited to making changes to the entire cornea [9]. A more targeted patient specific intervention in this disease has the potential to improve the treatment.

There has been some previous preliminary work in using OCT images as an input for modelling. One group studying acute angle-closure glaucoma (AACG) made measurements using OCT images and then used these to create a finite element model of the anterior chamber [10]. Their work managed to successfully create a model, which could give information about patients suffering from AACG. This shows the potential use for AS-OCT images in biomechanical modelling. However, their underlying program for the extraction of the structures of the anterior segment is not evaluated for accuracy.

The automated segmentation of the cornea in OCT images is a prerequisite step to derive the geometry of the cornea for biomechanical modelling. Of the currently commercially available OCT devices, only CASIA (Tomey, Tokyo, Japan) is able to both image and automatically segment the entire anterior segment [11]. However, we do not know the proprietary details of the segmentation approach used. The use of proprietary software only coming with the device will also limit the access for many applications. Other devices are either limited to only imaging a small section of the cornea or are unable to automatically segment the entire anterior segment like the Visante OCT system (Zeiss Meditec Inc., Dublin, California) [12]. The widespread use of OCT for biomechanical modelling requires imaging to be carried out by whatever device is present. Current automated measurement tools supplied with Visante OCT devices are limited to the central region of the cornea only, and manual measurement is time consuming, tedious and subject to human errors. For this reason, the development of fully automated segmentation techniques to accurately identify and trace both anterior and posterior boundaries of the anterior segment is required.

Segmentation of AS-OCT images has previously been carried out using a threshold-based technique [13], but this method was unable to accurately detect the posterior surface of the cornea. Work has been recently carried out using level set segmentation techniques on OCT images of the human cornea [14]. This technique achieved segmentation results comparable to those carried out by a manual observer. This method however, suffers from problems of relatively slow speed. Another group used graph theory and dynamic programming to segment a set of spectral domain OCT (SD-OCT) images of the central section of the cornea [15]. This method used the image gradient to identify edges on the images; Dijkstra’s algorithm [16] was then used to find a shortest path through the image based on the image gradient. Their results had good agreement with manual observers only for the central region of the cornea where the highest signal to noise ratio was found. Regions further away from the centre were approximated by fitting a polynomial curve to extend the surface. This method would not be very reliable if accurate information of a wider area of the cornea is wanted.

We propose a graph cut based segmentation technique for efficient and accurate segmentation of the cornea. Graph cuts are a way of segmenting an image exploiting a min-cut algorithm for graph labelling [17]. Graph cut methods have been used increasingly in image segmentation and other image processing problems. A set of pixels and a set of labels are input to the model and the goal is to minimize an energy function. A global optimum cut can be obtained for an image. Other groups have used graph cut segmentation to segment OCT images of the human retina [18,19]. The motivation of using this technique is that there are very efficient max flow algorithms that can be used to minimize the above energy [20]. In order to use graph cuts, an energy function must be constructed in the correct form [21]. There are many different terms that can be included into this function, such as terms based on regional statistics [22], and knowledge of shape [23].

In this paper, a new technique for segmentation of anterior and posterior surfaces of AS-OCT images is presented. Graph cut based segmentation is used to improve the speed and efficiency of the segmentation technique when compared to previously reported segmentation techniques. The results of the segmentation are evaluated over a data set, which has been segmented manually by an expert ophthalmologist.

## Methods

### Materials

39 AS-OCT B scan images through the centre of the cornea from healthy eyes (one image per eye) were used for the study. The study was approved by the Institutional Review Board and undertaken by following the tenets of the Helsinki Declaration. These images were acquired using the Visante AS-OCT system in Wenzhou Medical University, China. The Visante system is a time domain system that uses 1,300 nm infrared light to obtain cross-sectional images of the anterior segment with a scanning rate of 2,000 axial scans per second. Each B scan image contains 256 A-scans in 16 mm with 1024 points per A scan to a depth of 8 mm. The images have a transverse resolution of 60 μm and an axial resolution of 18 μm. The images were manually delineated by an expert ophthalmologist (FB) who marked the locations of the anterior and posterior boundaries.

### Automated segmentation

**Figure 1:**
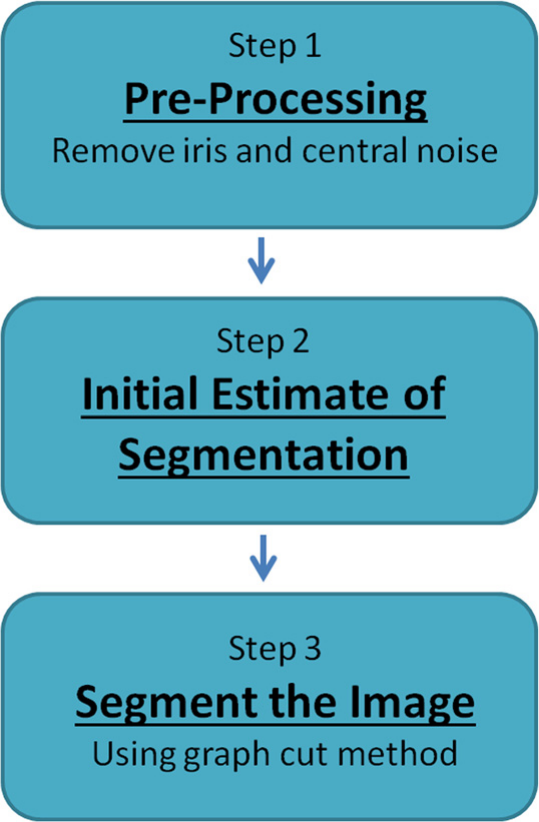
**Flow chart showing the three steps to the program.**

The method we have developed is a three-step process. Figure 1 is a flowchart showing the steps. The first step is to remove the iris from the image (since we are not attempting to segment it) and to remove noise artefacts from the image. The next step is to make an initial estimate of the segmentation using a threshold technique. The shape constraint will then be generated for the third step where graph cuts are used to create the final segmentation. Each of these steps will be discussed in more detail.

### Step 1: Removing iris and central noise

We have two structures present shown in our images; the cornea that is a curved structure and the iris that is largely horizontal in nature and has a gap in the centre. Both the iris and the cornea are defined by an increased intensity in the OCT images due to their dense structure scattering more light. This means that the iris has the potential to interfere with the detection of the cornea, so the exclusion of the iris from the image is desirable. OCT works by detecting scattered light. Since reflected light from the centre is of a much higher intensity than scattered light from within the cornea, most of OCT images will contain a bright, superior-inferior, narrow region at their centres. This region is an artefact due to the specula reflection of light from the centre where the cornea is perpendicular to the light source.

Both the iris and central noise can be detected and removed using a similar method. The central noise artefact is found by looking at the mean intensity of each column of pixels in the image. The columns with the maximum mean intensity correspond to the area of the central noise. Once this is detected, the intensities of the pixels within this region are all set to zero and excluded from the subsequent analysis. The same technique can be used for the detection of the iris. This time, the mean intensity of the rows is found. All rows below the horizontal line that is 10 pixels above the maximum intensity row are cropped out of the image. This line was empirically chosen since it resulted in the iris being cropped off in most images.

### Step 2: Initial segmentation using a threshold

The purpose of this step is to generate an approximate shape that is used to help guide the graph cut segmentation step. First, an entropy filter was applied to the image; this gave a measure of the texture of the image. The entropy image increased the contrast between the cornea and the background allowing a threshold to be used that would find most of the cornea. The value of the threshold was chosen using Otsu’s technique [24], which attempts to maximize variance between the two different classes while minimising the variance within each class at the same time. Once the threshold was chosen, all pixels with a value higher than the threshold were labelled as the cornea and the rest as part of the background.

An ellipse was then fitted to the anterior surface of the segmented shape. This was done since the cornea shape can be approximated as an ellipsoid. A related ellipse was then generated to give an initial estimate of the posterior surface since the threshold method could detect the anterior surface much better than the posterior surface. This second ellipse was generated by shrinking the first ellipse so that its top edge shifted by the distance of the central cornea width, which was calculated using an existing technique [25]. These two ellipses were then combined to generate an approximate shape for the cornea, which is used to help guide the next step of the segmentation.

### Step 3: Graph Cut Segmentation

The novel graph cut image segmentation program is used to segment the images. To use this to segment an image, we constructed a graph from the image. Each pixel on the image corresponds to a point on the graph. The values of the different points on the graph are controlled [26] to make the optimum path correspond to one that separates the cornea from the background to achieve segmentation.

Our segmentation method creates a function with three different terms to control the weighting of the points on the graph. The three terms used were a regional statistics term, a curvature term, and a shape term. The regional statistics term will act to segment the image according to the brightness of the pixels. It will find a segmentation that splits the light pixels from the dark pixels. Acting alone, this term will produce results similar to the threshold technique used in the initial step. The curvature term acts to keep the boundary smooth. It favours shorter boundaries by smoothing out any small boundary deviations due to image noise. The shape term is generated using the shape created in the second step to encourage the segmentation to be close to the boundaries of the prior shape. This helps the detection of parts of the cornea with low signal to noise ratio. Once the energy function was constructed, the minimum cut of this function could be found very quickly using the Dynamic Boykov-Kolmogorov algorithm [20].

### Evaluation

When testing the segmentation program, the strength of the regional and curvature terms were kept fixed. The optimum strength of the shape term was empirically found using a leave-one-out test for each image.

A comparison was carried out between the results of segmentation using this method and manual segmentation. Three different measures were used to evaluate the segmentation Dice’s similarity coefficient (DSC), mean unsigned surface positioning error (MSPE), and the Hausdorff distance (HD). DSC measures the overlap of two areas and gives a value of 1 for perfect agreement [27]. MSPE measures the unsigned distance between the boundaries from the program and manual segmentation. 95% HD measures the maximum distance between each point in one boundary and the corresponding point in the other. 95% HD excludes the longest 5% of the distances before finding the maximum distance between points in the reference and actual data [28].

A comparison of the results of different strengths of the shape constraint was carried out using the DSC. A comparison was also made with the results of a previous published level set based technique [14]. All segmentation was carried out using a Win7 PC with Intel Core i5-2320 CPU @ 3.00 GHz and 4.00 GB RAM. In order to test the significance of any differences, a paired t-test was carried out using SPSS (version 20, IBM). A p value of <0.05 is considered statistically significant.

## Results

The proposed method was applied to the entire data set of 39 images. Table 1 presents the results of segmentation using different values of the shape weighting term. The region term was always valued at 1 and the smoothing term at 0.2. The range of values of the shape term was chosen to cover the area with the best results. From Table 1, it can be seen that the shape weighting value of 0.1 performs best, thus this value was used throughout the rest of the analysis. Table 1 also shows the importance of using the shape term: a large drop in performance can be seen when a smaller shape term was used.

**Table 1:** **Dice’s similarity coefficient (DSC) comparison between segmentation results of graph cuts program with the manual segmentation over all the images**

**Value of shape (weighting term)**	0.01	0.05	0.1	0.2	0.3	0.4	0.5
**Mean**	0.501	0.896	0.943	0.933	0.923	0.912	0.906
**Standard deviation**	0.067	0.141	0.020	0.038	0.054	0.071	0.077

DSC is a dimensionless ratio where 1 corresponds to a perfect match between the images being compared.

**Figure 2:**
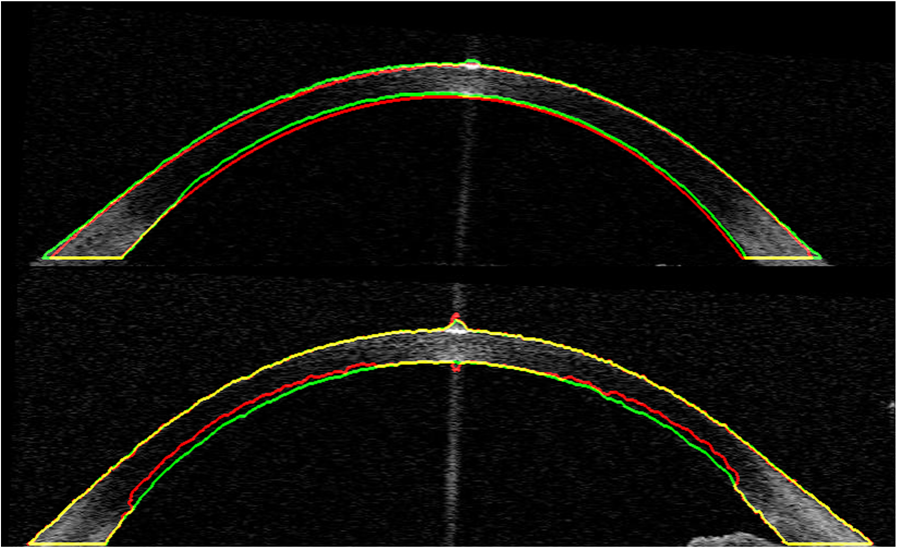
**Two example images with segmentation outlined.** New graph cut technique segmentation is marked using green line. Red line is segmented using level set technique. Yellow lines indicate where the two segmentation methods agree.

Figure 2 shows an example of image segmentation. The results of two different segmentation methods are shown. The segmentation accuracy measured by the DSC, MSPE and HD is presented in Table 2. The method developed here and an existing level set method were compared with manual segmentation. A series of four paired t-tests was carried out to compare the results. The DSC results show that the new graph cut technique performs significantly better than the previously used level set technique (p < 0.05). Looking at the results for the MSPE of both surfaces, a significant improvement in accuracy for the new method can be seen (p < 0.05 for both surfaces). The results for the posterior surface are not as good as those for the anterior surface, this is due to the lower signal to noise ratio for parts of the posterior surface. The newly developed graph cut technique also showed a significant improvement in HD when compared to the results of the existing level set technique (p < 0.05).

**Table 2:** **Comparison between segmentation results and manual segmentation**

**Segmentation technique**	**Graph cut**	**Level set**
**DSC**		
Mean	0.943	0.919
Standard deviation	0.020	0.026
**MSPE- anterior**		
Mean	1.21	1.64
Standard deviation	0.31	0.52
**MSPE - posterior**		
Mean	2.82	3.90
Standard deviation	1.29	1.81
**95% HD**		
Mean	6.66	9.50
Standard deviation	2.72	4.33

Two techniques are shown, the graph cut program described here, and a previously published level set technique. Four different methods are used to measure the difference between techniques. All measurements are given in terms on pixels on an image. DSC: Dice’s similarity coefficient, MSPE: mean unsigned surface positioning error, HD: Hausdorff distance.

Table 3 compares the running time of the two methods. The time to run the graph cut program is substantially shorter than the level set method [14], thus achieving a reduction in segmentation time by a factor of 50. Both methods are faster than manual segmentation, which took approximately 15 minutes per image. A full statistical analysis was not performed due to the clear difference between the two segmentation methods.

**Table 3:** **Mean time taken for segmentation using graph cut method (GC) and level set method (LS)**

**Time (s)**	**GC**	**LS**
**Mean**	2.53	131.4
**Standard deviation**	0.65	38.5

The graph cut method shows a significant reduction in time needed for segmentation.

## Discussion

A new segmentation technique for anterior segmentation OCT images has been developed. The technique has demonstrated high-speed segmentation with an average segmentation time of 2.53 seconds per image. The segmentation technique has shown comparable accuracies to those achieved using manual segmentation in a fraction of the time it would take to manually segment an image.

Our new technique was compared to a previously published level set based technique. Using four different comparison methods, the newly developed method was shown to be significantly better. Another major advantage of our new method is the speed of the technique. The technique presented here is over 50 times faster. This difference is very important when looking at practical applications of the technology. If the technique is to be useful as a diagnostic tool, it is essential to process the image fast enough, so that it can be completed while the patient is still present.

Accurate segmentation of the cornea is important for analysis of structures further into the eye. Light will experience refraction when it passing through the cornea, which will affect the detection of other structures in the image. Since our focus of segmentation was to investigate the cornea, we have not carried out any correction of the image. If accurate information on the iris was required, then this method could be used to calculate the distortion that the cornea will cause to the rest of the image, allowing a correction to be made.

Further improvements to the model could be made by carrying out further investigations into the strength of the shape term to use over a larger data set. A different method of representing the shape could produce improved results for this technique, such as the use of a statistical shape model [29] created using a training set of manually segmented images. While this method has shown significant increases in speed compared to previous techniques, it might be possible to further increase the speed by a process of code optimisation. This segmentation has been carried out using a combination of Matlab and C++. Exclusive use of C++ may enable further speedup.

Another improvement that could be made is to alter the step involving removal of the iris. Currently, the step works by removing an entire section of the image where the iris is present. In cases where the iris is deformed, such as by iris tumours or other problems, this may result in too large a section of the image being removed. One solution to this would be to use a more advanced iris removal technique. For example, we have previously demonstrated that segmentation of the iris is much easier than segmentation of the cornea [14]. Segmentation of the iris could be used to remove only the iris from the part of the image, which it is present in.

Future work on this technique will focus on expanding the segmentation technique to full 3D segmentation. This will then enable our segmentation to be used to create patient specific biomechanical models of the cornea.

## Conclusions

This is the first time graph cut segmentation has been demonstrated for use on AS-OCT images. The method has been demonstrated to be faster than previous techniques with improved accuracy. The speed of this technique means it can be used to segment images while a patient waits and allow for rapid interpretation of the images. This technique can thus be used to produce an input for patient specific biomechanical models of the human eye.

## Appendix: segmentation energy function

In order to achieve accurate segmentation of AS-OCT images, an energy function including both shape- and image-based terms was used. A binary shape term was used. The energy function is given by

1 $$ E={\displaystyle \sum_i}\left({\lambda}_{image}{\left({u}_i-{c}_1\right)}^2+{\lambda}_{shape}M(p)\right){x}_i+\left({\lambda}_{image}{\left({u}_i-{c}_2\right)}^2-{\lambda}_{shape}M(p)\right)\left(1-{x}_i\right)+\mu {\displaystyle \sum_{k=1}^n}{\displaystyle \sum_{\left(i,j\right)}}{\omega}_k\left(\left(1-{x}_i\right){x}_j+{x}_i\left(1-{x}_j\right)\right) $$

Where *λ*_*image*_, and *λ*_*image*_ are coefficients determining the strength of image and shape terms respectively, *u*_*i*_ is image intensity at point *i,* c_1_ is mean intensity of object, c_2_ mean intensity of background, *x*_*i*_ is binary function that determines if point *i* is part of the object or background and *M (p)* is a signed distance function defined by the shape that guides the segmentation.
